# Non‐motor phenotypic subgroups in adult‐onset idiopathic, isolated, focal cervical dystonia

**DOI:** 10.1002/brb3.2292

**Published:** 2021-07-21

**Authors:** Megan E. Wadon, Grace A. Bailey, Zehra Yilmaz, Emily Hubbard, Meshari AlSaeed, Amy Robinson, Duncan McLauchlan, Richard L. Barbano, Laura Marsh, Stewart A. Factor, Susan H. Fox, Charles H. Adler, Ramon L. Rodriguez, Cynthia L. Comella, Stephen G. Reich, William L. Severt, Christopher G. Goetz, Joel S. Perlmutter, Hyder A. Jinnah, Katharine E. Harding, Cynthia Sandor, Kathryn J. Peall

**Affiliations:** ^1^ Neuroscience and Mental Health Research Institute Division of Psychological Medicine and Clinical Neurosciences Cardiff University Maindy Road Cardiff CF24 4HQ UK; ^2^ Institute of Neurology University College London Queen Square London WC1N 3BG UK; ^3^ School of Medicine Cardiff University Heath Park Campus Cardiff CF14 4YS UK; ^4^ Division of Neurology University of British Columbia Wesbrook Mall Vancouver British Columbia V6T 2B5 Canada; ^5^ Department of Neurology University of Rochester Elmwood Avenue Rochester New York NY 14642 USA; ^6^ Menninger Department of Psychiatry Baylor College of Medicine Butler Boulevard Houston Texas 77030 USA; ^7^ Departments of Neurology & Human Genetics Emory University Woodruff Circle Atlanta Georgia 30322 USA; ^8^ Edmond J Safra Program in Parkinson Disease Movement Disorder Clinic Toronto Western Hospital Bathurst Street Toronto Ontario M5T 2S8 Canada; ^9^ Department of Medicine University of Toronto Queen’s Park Crescent West Toronto Ontario M5S 3H2 Canada; ^10^ The Parkinson's Disease and Movement Disorders Center Mayo Clinic Department of Neurology East Shea Boulevard Scottsdale Arizona 85259 USA; ^11^ Department of Neurology University of Florida Newell Drive Gainesville Florida 32611 USA; ^12^ Department of Neurological Sciences Rush University Medical Center West Harrison Street Chicago Illinois 60612 USA; ^13^ Department of Neurology University of Maryland School of Medicine south Paca Street Baltimore Maryland 21201 USA; ^14^ Beth Israel Medical Center First Avenue New York New York 10003 USA; ^15^ Neurology Radiology Neuroscience Physical Therapy and Occupational Therapy Washington University School of Medicine South Euclid Avenue St. Louis Missouri 63110 USA; ^16^ Department of Neurology Aneurin Bevan University Health Board Corporation Road Newport NP19 0BH UK; ^17^ UK Dementia Research Institute Cardiff University Maindy Road Cardiff CF24 4HQ UK

**Keywords:** dystonia disorders, phenotype, surveys and questionnaires, torticollis

## Abstract

**Background**: Non‐motor symptoms are well established phenotypic components of adult‐onset idiopathic, isolated, focal cervical dystonia (AOIFCD). However, improved understanding of their clinical heterogeneity is needed to better target therapeutic intervention. Here, we examine non‐motor phenotypic features to identify possible AOIFCD subgroups.

**Methods**: Participants diagnosed with AOIFCD were recruited via specialist neurology clinics (dystonia wales: *n* = 114, dystonia coalition: *n* = 183). Non‐motor assessment included psychiatric symptoms, pain, sleep disturbance, and quality of life, assessed using self‐completed questionnaires or face‐to‐face assessment. Both cohorts were analyzed independently using Cluster, and Bayesian multiple mixed model phenotype analyses to investigate the relationship between non‐motor symptoms and determine evidence of phenotypic subgroups.

**Results**: Independent cluster analysis of the two cohorts suggests two predominant phenotypic subgroups, one consisting of approximately a third of participants in both cohorts, experiencing increased levels of depression, anxiety, sleep impairment, and pain catastrophizing, as well as, decreased quality of life. The Bayesian approach reinforced this with the primary axis, which explained the majority of the variance, in each cohort being associated with psychiatric symptomology, and also sleep impairment and pain catastrophizing in the Dystonia Wales cohort.

**Conclusions**: Non‐motor symptoms accompanying AOIFCD parse into two predominant phenotypic sub‐groups, with differences in psychiatric symptoms, pain catastrophizing, sleep quality, and quality of life. Improved understanding of these symptom groups will enable better targeted pathophysiological investigation and future therapeutic intervention.

## Graphical abstract

We demonstrate that individuals with adult‐onset idiopathic, isolated, focal cervical dystonia parse into two subgroups dependent on their non‐motor symptoms. Approximately a third of participants showed increased levels of depression, anxiety, sleep impairment and pain catastrophising, as well as decreased quality of life, indicating that improved understanding of these symptom groups will lead to better targeted treatment plans.

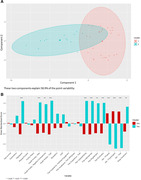

## 1 INTRODUCTION

Dystonia involves co‐contraction of antagonistic muscles leading to abnormal postures and movements. This causes considerable disability, impacting social interaction, education and health economic outcomes. Adult‐onset idiopathic, isolated, focal cervical dystonia (AOIFCD) is the most common form of adult onset dystonia encountered in neurological practice, with an estimated prevalence of 4.98 per 100,000 and onset typically in the fifth decade of life (Steeves et al., 2012). Patients most commonly present to clinical services due to their motor symptoms, although increasing evidence suggests a significant non‐motor phenotype to be associated with most forms of dystonia, including AOIFCD (Berman et al., 2017; Conte et al., 2016; Peall et al., 2015). Debate persists as to whether these non‐motor symptoms represent a primary component of the disorder phenotype, or secondary sequelae to a chronic disorder.

Several cohort studies have demonstrated a consistent psychiatric phenotype, with symptoms reported in up to 65.9% of individuals, the most common of which being major depressive disorder and anxiety related disorders (Moraru et al., 2002; Peall et al., 2013; Wenzel et al., 1998). These symptoms have also been shown to impact quality of life (QoL), sometimes to a greater extent than that of the motor symptom severity (Ben‐Shlomo et al., 2002; Müller et al., 2002; Page et al., 2007). Obsessive‐compulsive symptoms are also reported at higher rates amongst those with primary focal dystonia, compared to control cohorts, although symptoms are frequently under diagnosed, largely untreated, and with no standardized treatment model (Barahona‐Corrêa et al., 2011; Govoni et al., 2001).

Although fewer studies have focused on this, pain is the most commonly reported non‐motor symptom in cervical dystonia (54.6–88.9%) (Camargo et al., 2015; Charles et al., 2014; Tassorelli et al., 2006; Williams et al., 2017). Several models have been proposed for this, including the prolonged muscular contraction, changes to nociceptive pathways reducing pain thresholds, and changes in the cortical somatosensory system (Lobbezoo et al., 1996; Tinazzi et al., 2003; Tinazzi et al., 2012). Finally, sleep difficulties have also been identified in those with AOIFCD. These include poorer sleep quality, impaired sleep architecture, increased fatigue, and excessive daytime sleepiness, independent of motor severity (Antelmi et al., 2017; Marenka Smit et al., 2017). Interestingly excessive daytime sleepiness and sleep quality have also been associated with psychiatric comorbidities and reduced QoL (Marenka Smit et al., 2017).

Previous studies have tended to involve case‐control comparison, often focusing on specific groups of non‐motor symptoms in small cohorts (Ben‐Shlomo et al., 2002; Girach et al., 2019; Marenka Smit et al., 2017; Tomic et al., 2016; van den Dool et al., 2016). In addition, studies have often considered AOIFCD to be a homogenous disorder, in spite of the significant phenotypic variability often observed in clinical practice. Here we have sought to address both of these elements, examining a more comprehensive spectrum of non‐motor symptoms (psychiatric symptoms, pain, and sleep disturbance) aimed at investigating evidence of phenotypic sub‐grouping across two independently recruited cohorts.

## 2 METHODS

### 2.1 Participants

Data from individuals diagnosed with AOIFCD were collected from two cohorts. The dystonia wales cohort involved those recruited in specialist neurology clinics in Cardiff. All participants were >18 years of age and able to provide informed consent (REC ref: 14/WA/0017, IRAS ID: 146495 and REC ref: 18/WM/0031, IRAS ID: 236219). The second cohort involved those recruited to the Natural History Project database of the dystonia coalition (https://www.rarediseasesnetwork.org/cms/dystonia) via specialist neurology clinics and included only those with a clinical diagnosis of AOIFCD enrolled at specialist centers in North America between 16^th^ March 2011 and 31^st^ January 2013. For both cohorts, participants were only included if they did not have a diagnosis of another movement disorder and had not been treated with deep brain stimulation.

### 2.2 Data collection

Participants in both studies underwent an extensive battery of non‐motor assessments covering psychiatric health, pain, sleep difficulties, and QoL (Table 1).

**TABLE 1:** The questionnaires used in the assessment of non‐motor symptoms for all participants

**Assessment**	**Outcome measure**	**Dystonia coalition cohort**	**Dystonia wales cohort**
**Psychiatric**			
Becks depression inventory^26^	Depression	✓	✓
Patient health questionnaire 9 (PHQ9)^33^	Depression	✓	
Health anxiety inventory (HAI)^27^	Health anxiety Overall, negative consequences		✓
Liebowitz social anxiety scale (LSAS)^36^	Social anxiety Fear Avoidance	✓	
Hospital anxiety and depression scale (HADS)^34^	Mixed depression and anxiety	✓	
TWSTRS mood and anxiety score^35^	Mixed depression and anxiety	✓	
Yale‐brown obsessive‐compulsive scale (YBOCS)^28^	Obsessions, compulsions, and obsessive‐compulsive disorder		✓
SCID‐I^37^	Axis 1 psychiatric disorders	✓	
**Pain**			
TWSTRS pain scale^35^	Pain severity, pain duration, pain disability, total pain	✓	
Pain catastrophizing scale (PCS)^29^	Pain catastrophizing, rumination, magnification, helplessness		✓
Chronic pain acceptance questionnaire (CPAQ)^30^	Pain acceptance, activities engagement, pain willingness		✓
**Sleep**			
Pittsburgh sleep quality index (PSQI)^31^	Sleep impairment		✓
**Disability**			
TWSTRS disability scale^35^	Disability	✓	
**Quality of life**			
Short form‐36 health survey (SF‐36)^32^	Overall quality of life, psychological quality of life, physical quality of life	✓	✓

The two right hand columns indicate which assessments were used in which cohorts.

SCID‐I, Structured Clinical Interview for DSM‐IV Axis I Disorders; TWSTRS, Toronto Western Spasmodic Torticollis Rating Scale.

### 2.3 Dystonia wales cohort

Data was collected using a series of self‐complete questionnaires. Participants were able to complete these questionnaires either online, using the study specific website, or in paper format. Demographic and clinical data collected included age, sex, age at onset, and family history of dystonia. Systematic and standardized questionnaires were used to assess non motor symptoms including:

- Psychiatric health—Beck's depression inventory (BDI) (Dozois et al., 1998), health anxiety inventory (HAI) (Salkovskis et al., 2002), and the Yale‐brown obsessive compulsive scale (YBOCS) (Goodman et al., 1989).
- Pain—Pain catastrophizing scale (PCS) (Sullivan et al., 1995) and chronic pain acceptance questionnaire (CPAQ) (McCracken et al., 2004). Participants were also asked if they suffered from pain due to AOIFCD.
- Sleep difficulties—Pittsburgh sleep quality index (Buysse et al., 1989).
- QoL—Short form‐36 health survey (SF‐36) (Ware, 1994).

### 2.4 Dystonia coalition cohort

Participants attended face‐to‐face interviews where non‐motor assessments included:

- Psychiatric health—BDI (Dozois et al., 1998), patient health questionnaire 9 (PHQ9) (Kroenke et al., 2001), hospital anxiety and depression scale (HADS) (Zigmond & Snaith, 1983), Toronto western spasmodic torticollis rating scale (TWSTRS) Mood and Anxiety Score (Consky et al., 1990), Liebowitz social anxiety scale (LSAS) (Heimberg et al., 1999), and structured clinical interview for DSM‐IV Axis I disorders (First et al., 1997).
- Pain—TWSTRS pain scale (Consky et al., 1990)
- Disability—TWSTRS disability scale (Consky et al., 1990)
- QoL—Short form‐36 health survey (SF‐36) (Ware, 1994).

Demographic and clinical data were also collected for all participants.

### 2.5 Independent analysis of dystonia wales and dystonia coalition cohorts

All analysis was performed using R version 3.6.3 (Team, 2020). The following analyses were undertaken independently in the Dystonia Wales and Dystonia Coalition cohorts due to the distinct questionnaires used, albeit addressing common symptom themes. These included: i) k‐means cluster analysis and ii) Bayesian multiple phenotype mixed model (BMPMM) analysis, aimed at exploring variation in clinical phenotypes within AOIFCD populations. For the cluster analysis, within each cohort all scores were standardized and the pre‐defined optimal number of clusters determined using the gap statistic, silhouette, and elbow methods, before k‐means clustering was performed using the Hartigan‐Wong algorithm (Hartigan & Wong, 1979). Participants with missing data for any variable were excluded from the cluster analysis, therefore only participants with complete datasets were included. The number of participants in each cluster was determined and standardized scores were compared between the clusters to determine the differences. All *p*‐values were corrected for multiple comparisons using the Bonferroni multiple comparison adjustment method.

The PHENIX package in R was used for the BMPMM approach (Dahl et al., 2016), removing variables where >25% of participants included in the analysis were missing data for that variable (no questionnaire/variable score available), and determining the phenotypic axes that explain the greatest proportion of phenotypic variation in each cohort. If participants had no data available for multiple questionnaires and had data missing for an entire non‐motor category (e.g., pain), that participant was removed from this analysis. Correlation analyses determined the clinical features each phenotypic axis represented. The primary axes for each cohort were compared to determine if they represented the same phenotypic subgroup. Clinical variables were assigned to one of 8 phenotypic categories: “Onset,” “Disease Duration,” “Gender,” “Family history,” “Pain,” “Anxiety/Depression,” “Other Psychiatric,” or “Other.” The “Other” category included clinical characteristics that were only measured in one cohort (including sleep impairment, disability, and if a sensory trick was effective in relieving symptoms) and clinical characteristics that were excluded in one cohort due to missing data (QoL). For each cohort the mean correlation by phenotypic category was computed and axes compared using the mean correlation for each phenotypic category.

## 3 RESULTS

### 3.1 Demographic characteristics

One hundred and fourteen participants diagnosed with AOIFCD from the UK (78F, 31M, 5 not declared) were recruited. Median age at dystonia symptom onset was 44 years (inter‐quartile range 37–54 years) and 64 years (inter‐quartile range 57–71 years) at examination. One hundred and eighty‐three individuals diagnosed with AOIFCD (134F, 49M) were recruited to the dystonia coalition cohort (Comella et al., 2015, 2016) with a median age at motor symptom onset of 45 years (inter‐quartile range 37–54 years) and median age at examination of 60 years (inter‐quartile range 54–67.5 years). Table 2 summarizes the demographic and clinical characteristics of both cohorts.

**TABLE 2:** Demographic characteristics for dystonia coalition and dystonia wales cohorts

	**Dystonia coalition cohort**			**Dystonia wales cohort**		
	**Complete cohort**	**Included in cluster analysis**	**Included in BMPMM analysis**	**Complete cohort**	**Included in cluster analysis**	**Included in BMPMM analysis**
Number of participants	183	32	183	114	43	75
Gender						
Female	134 (73.2%)	19 (59.4%)	134 (73.2%)	78 (68.4%)	33 (76.7%)	52 (69.3%)
Male	49 (26.8%)	13 (40.6%)	49 (26.8%)	31 (27.2%)	10 (23.3%)	18 (24.0%)
Not declared	0	0	0	5 (4.4%)	0	5 (6.7%)
Age at examination (Median, IQR)	60 (54–67.5)	56 (51.75–63)	60 (54–67.5)	64 (57–71)	62 (54.5–71)	64 (56.25–71.75)
Age at onset of motor symptoms (Median, IQR)	45 (37–54)	48 (41.75–53)	45 (37–54)	45 (33–52.25)	45 (33.5–53.5)	44 (34.5–53)
Disease duration (Years) (Median, IQR)	13 (6–21)	8.5 (3–16.5)	13 (6–21)	17 (9.75–25.25)	13 (7–24)	16 (9–25.5)
Treatment with botulinum toxin						
Yes	‐	‐	‐	94 (82.5%)	35 (81.4%)	61 (81.3%)
No	‐	‐	‐	16 (14.0%)	7 (16.3%)	11 (14.7%)
Not specified	‐	‐	‐	4 (3.5%)	1 (2.3%)	3 (4.0%)

BMPMM, Bayesian multiple phenotype mixed model; IQR, Inter‐Quartile Range.

‐ Indicates that this information was not available.

### 3.2 Cluster analysis

**FIGURE 1:**
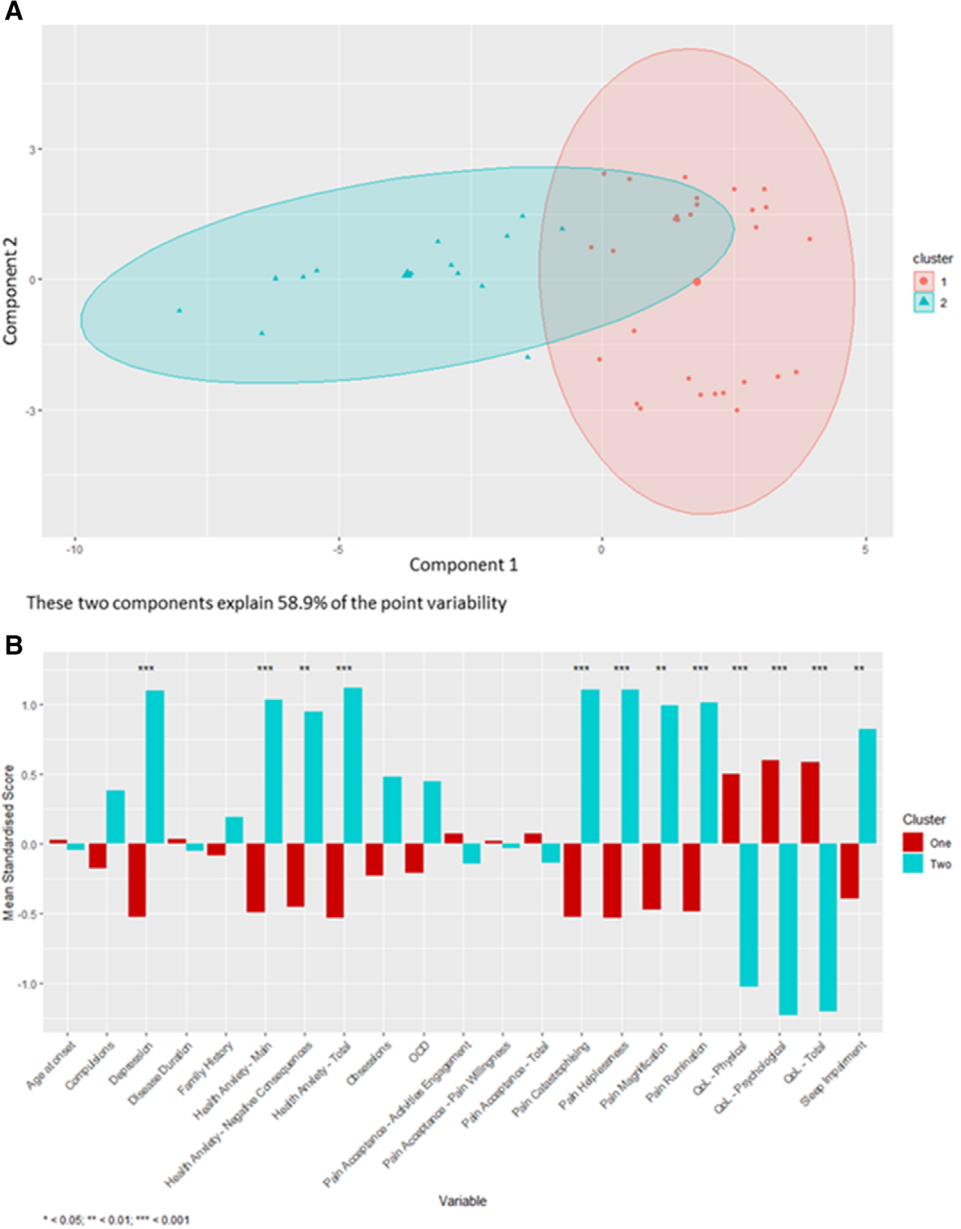
The results of the cluster analysis for the dystonia wales cohort. A) A schematic visualization of clustering as calculated in the k‐means cluster analysis. Component 1 and component 2 represent the two principal components that represent that greatest amount of point variability. B) A comparison of the mean standardized scores for each of the variables measured in the dystonia wales cohort between the participants in allocated to cluster one and cluster two in the cluster analysis. Abbreviations: BDI, Beck Depression Inventory; HAI, Health Anxiety Inventory; QoL, Quality of life

**I**n the Dystonia Wales cohort, after excluding participants with any missing data, 43 participants with complete datasets were included in the analysis. The optimal number of clusters was determined to be two, with 29 individuals in the first cluster and 14 individuals in the second cluster (Figure 1(A)). Significant differences between the two clusters occurred in psychiatric, pain, sleep, and QoL scores (Figure 1(B)). Participants in Cluster two showed significantly higher scores for depression (*p* < 0.001), total health anxiety (*p* < 0.001), main health anxiety, and the negative consequences of health anxiety (*p* < 0.001 and *p* < 0.01, respectively). Significantly higher levels of pain catastrophizing (*p* < 0.001), including helplessness, magnification, and rumination (*p* < 0.001, *p* < 0.01, and *p* < 0.001, respectively), increased sleep impairment (*p* < 0.01) lower QoL scores (*p* < 0.001) were also noted in cluster two compared to cluster one.

**FIGURE 2:**
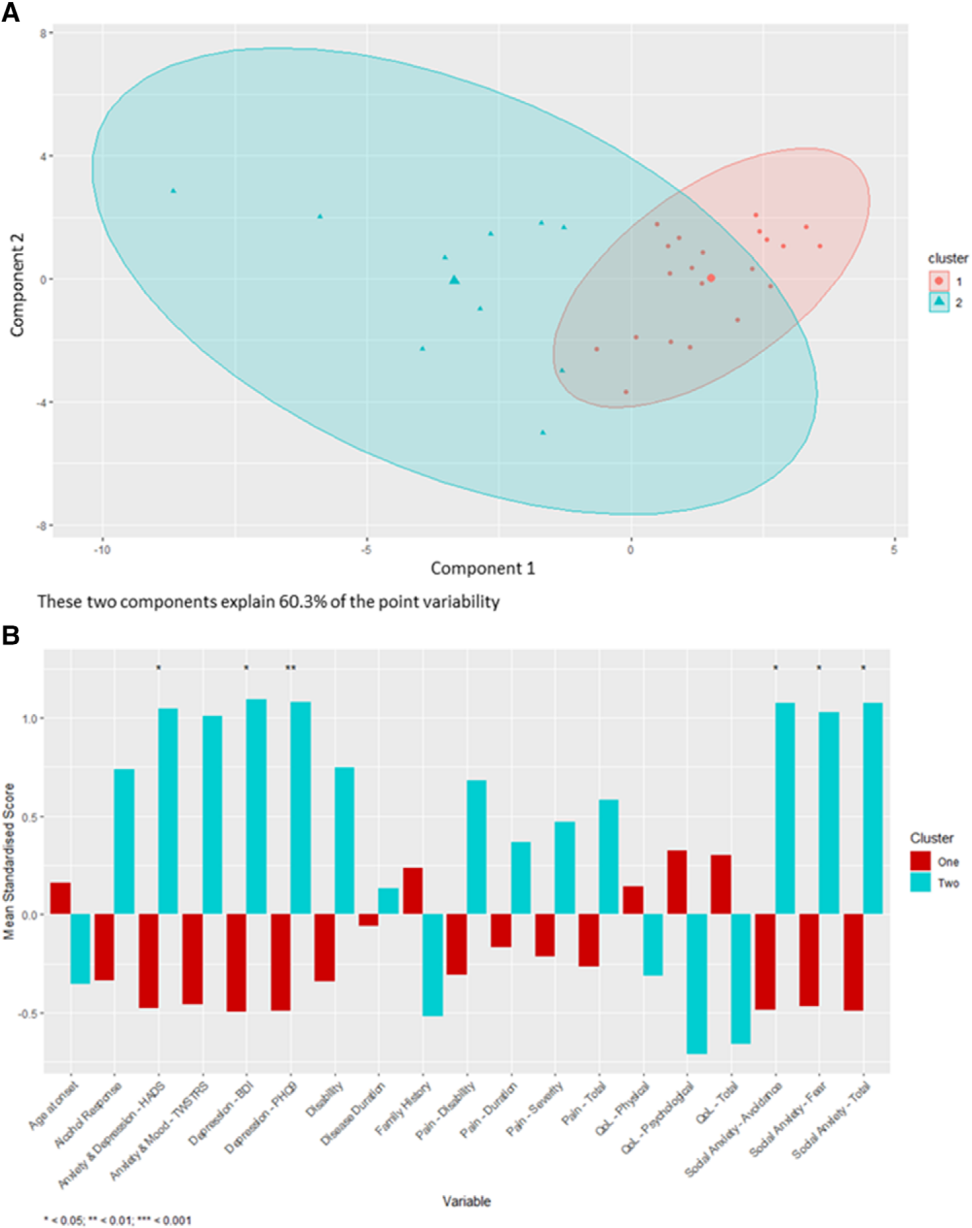
The results of the cluster analysis for the dystonia coalition cohort. A) A schematic visualization of clustering as calculated in the k‐means cluster analysis. Component 1 and component 2 represent the two principal components that represent that greatest amount of point variability. B) A comparison of the mean standardized scores for each of the variables measured in the dystonia coalition cohort between the participants in allocated to cluster one and cluster two in the cluster analysis. Abbreviations: BDI, Beck Depression Inventory; HADS, Hospital Anxiety and Depression Scale; LSAS, Liebowitz Social Anxiety Scale; PHQ9, Patient Health Questionnaire 9; QoL, Quality of life; TWSTRS, Toronto Western Spasmodic Torticollis Rating Scale

Thirty‐two participants with complete datasets were included in the dystonia coalition cluster analysis. The optimal number of clusters was determined to be two, with 22 participants forming the first cluster and 10 participants forming the second cluster (Figure 2(A)). In this cohort, significant differences between the two clusters were restricted to psychiatric measures, with depression, as measured by the BDI and PHQ9 (*p* < 0.05 and *p* < 0.01, respectively), HADS anxiety and depression score (*p* < 0.05), and social anxiety (*p* < 0.05), including avoidance due to social anxiety and fear due to social anxiety (*p* < 0.05 and *p* < 0.05, respectively) being significantly higher in cluster 2 compared to cluster 1 (Figure 2(B)). Cluster two also demonstrated higher scores for disability, disability due to pain, total pain, TWSTRS Mood and Anxiety score, and response to alcohol, as well as decreased QoL scores compared to cluster one, however these differences did not reach the Bonferroni adjusted threshold for statistical significance.

### 3.3 Bayesian multiple phenotype mixed model

**FIGURE 3:**
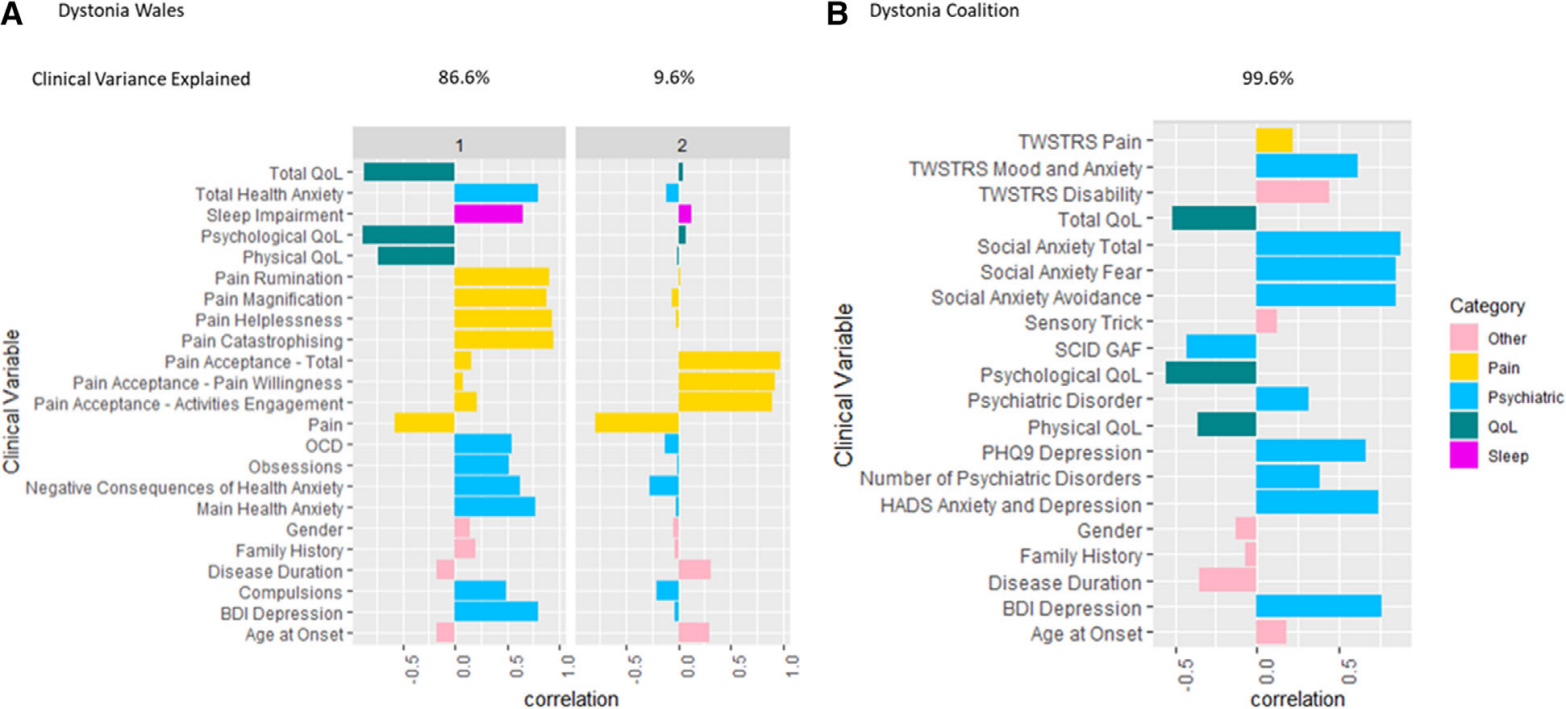
The correlations between clinical variables and the phenotypic axes derived using a bayesian multiple phenotype mixed model in A) the dystonia wales cohort and B) the dystonia coalition cohort. Clinical variables are divided into phenotypic categories. Abbreviations: BDI, Beck's Depression Inventory; GAF, Global Assessment of Functioning; HADS, Hospital Anxiety and Depression Scale; OCD, Obsessive‐compulsive disorder; PHQ9, Patient Health Questionnaire 9; QoL, Quality of life; SCID, Structured Clinical Interview for DSM‐IV Axis I Disorders; TWSTRS, Toronto Western Spasmodic Torticollis Rating Scale

Two main phenotypic axes were derived from the Dystonia Wales cohort (*n* = 75), explaining 86.6% and 9.6% of the observed clinical variance respectively (Figure 3(A)). Axis 1 predominantly identified increased psychiatric symptomatology, including health anxiety, OCD, depression, and pain interpretation (rumination, magnification, helplessness, and catastrophizing), coupled with lower QoL and pain. Axis 2 was most strongly associated with higher pain levels and lower levels of pain acceptance. Within the dystonia coalition cohort (*n* = 183) QoL scores were removed when deriving the phenotypic axes due to >25% of participants not having QoL data available. This resulted in one phenotypic axis, explaining >99% of the observed clinical variance (Figure 3(B)). QoL was then re‐introduced to determine whether these scores have the potential to align with the predetermined axis. The phenotypic axis was most strongly associated with increased psychiatric symptomatology (anxiety, depression, and social anxiety), as well as decreased QoL.

**FIGURE 4:**
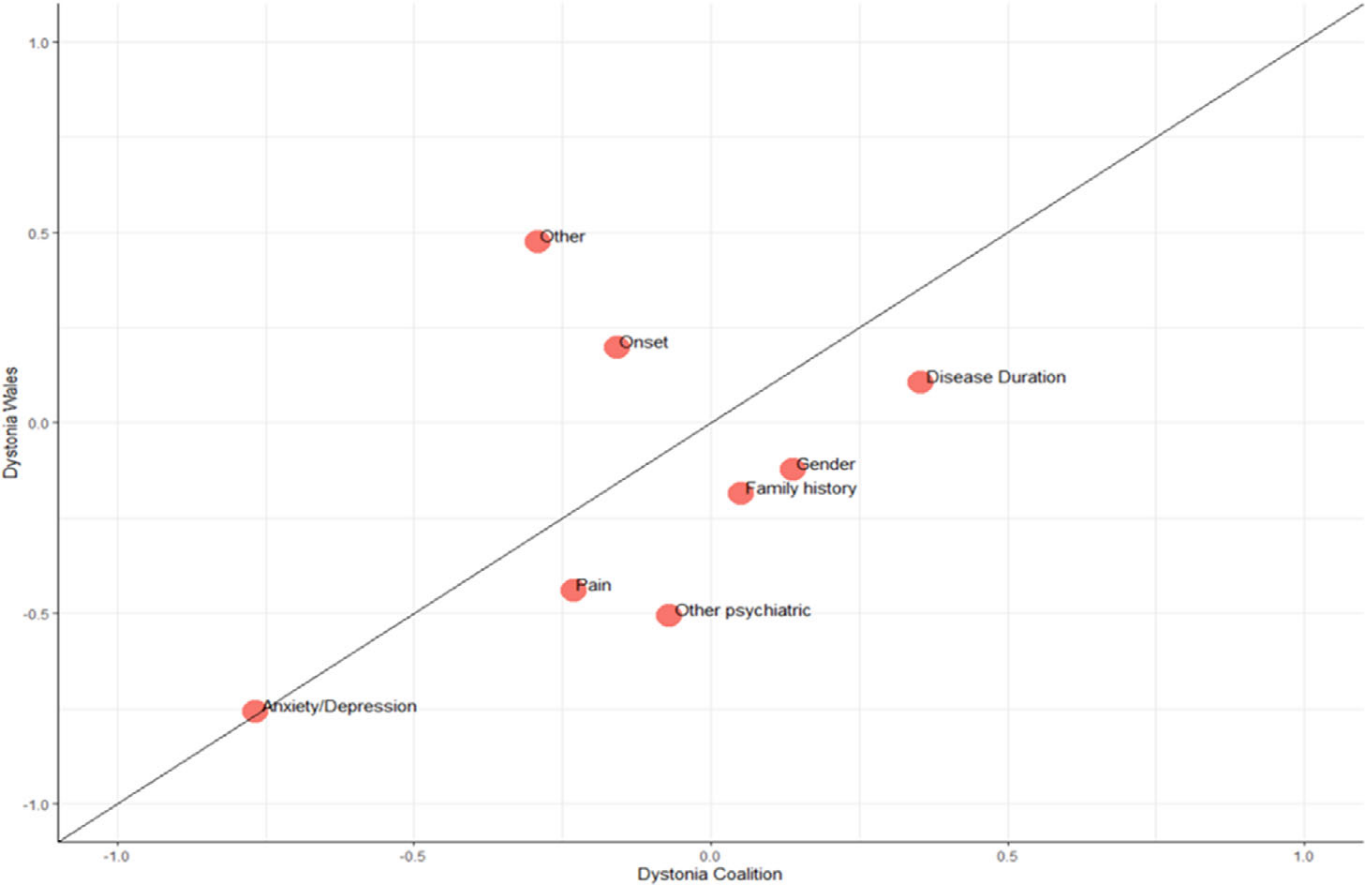
The relationship between Axis 1 in the dystonia wales cohort and the phenotypic axis in the dystonia coalition cohort determined by the bayesian multiple phenotype mixed model analysis for different categories of variable

The axis that explained the highest level of clinical variation in the dystonia wales cohort (Axis 1) was compared to the axis produced in the dystonia coalition cohort to determine their degree of similarity using eight variable categories that represented the common clinical themes in each dataset. Variables were included in the categories if they had been used to derive the phenotypic axes for the corresponding cohort. These included: onset (age at dystonia onset), disease duration, gender, family history of dystonia, pain (presence of pain, PCS, CPAQ, TWSTRS pain), anxiety/depression (BDI, HAI, TWSTRS mood and anxiety, PHQ9, HADS, LSAS), other psychiatric (YBOCS, presence of a psychiatric disorder, number of psychiatric disorders, global assessment of functioning), other (TWSTRS disability, QoL, sensory trick, sleep impairment). The “Other” category contained variables that were either only available in one cohort or were excluded when deriving the phenotypic axes, for example, QoL in deriving axes for the dystonia coalition cohort. When all categories were included there was no significant correlation between the cohorts (*r* = 0.43, *p* = 0.29, Figure 4). As the “Other” category varies greatly between the two cohorts and “Onset” typically represented onset of motor symptoms, which did not cluster with non‐motor symptoms in the cluster analysis and had no strong relationships with the axes in the BMPMM analysis, we conducted this analysis again with these categories removed to determine if this would make the relationship stronger. With the “Onset” and “Other” categories removed, this resulted in the primary axes being significantly similar across the two cohorts (*r* = 0.94, *p* = 0.005).

## 4 DISCUSSION

This study encompasses broad non‐motor symptom phenotyping in two independent AOIFCD cohorts to evaluate evidence of non‐motor phenotypic subgrouping that may account for the symptomatic heterogeneity observed in clinical practice. This is especially important, as previous studies have investigated motor phenotypic subgroups in related disorders (Defazio et al., 2017; Shaikh et al., 2020), but non‐motor features have not previously been considered. Cluster analysis demonstrated similar patterns of psychiatric symptoms (anxiety, depression, pain catastrophizing) and reduced QoL clusters. Generalized linear mixed modelling (BMPMM) further supported this with psychiatric symptomatology accounting for the majority of the clinical variance observed in both cohorts. Age at onset of the motor symptoms, family history of a similar motor disorder, disease duration, and sex were not strong determining components of any of the phenotypic axes identified and did not differ significantly between the clusters identified in the cluster analysis in either the dystonia wales or the dystonia coalition cohorts. Overall, these data suggest that psychiatric symptomatology contribute more to the AOIFCD phenotypic spectrum than other non‐motor symptoms, but that the balance and proportions of these symptoms may differ between phenotypic subgroups. The low level of association of different subgroups with disease duration lends some support that these symptoms represent primary phenotypic components of the AOIFCD symptom spectrum, rather than secondary sequelae to chronic disease.

Previous studies have identified increased psychiatric symptoms in AOIFCD, the most prominent of which being anxiety and depression (Moraru et al., 2002; Peall et al., 2013; Wenzel et al., 1998). We identified psychiatric symptoms as being key contributors to the phenotypic subgroups identified during cluster analysis with anxiety and depression being significantly different between clusters in both the dystonia wales and dystonia coalition cohorts, with approximately a third of patients experiencing increased anxiety and depression. This was consistent with the results using the Bayesian approach in which phenotypic axes explaining the most variance in both cohorts (axis 1) involved a psychiatric predominant phenotype, which also included a higher level of perception of the negative aspects of pain in the Dystonia Wales cohort. Greater detriment to QoL also appears to be consistently reported in this subgroup, supporting the findings from previous studies that have shown psychiatric symptomatology to have a greater impact on QoL than motor symptoms (Smit et al., 2016). Participants in cluster two also demonstrated higher levels of sleep impairment compared to cluster one, as well as a suggested association using the Bayesian approach of sleep impairment, depression and anxiety (Dystonia Wales Cohort). This builds on prior research which has found increased sleep difficulties in AOIFCD, as well as, sleep difficulties being associated with increased psychiatric comorbidities and reduced QoL (Antelmi et al., 2017; Smit et al., 2017).

High levels of reported pain in AOIFCD is well established (Avenali et al., 2018) however, few previous studies have explored the perception, interpretation and attitudes towards pain within this clinical group. While the cluster analysis suggested that pain acceptance was comparable between AOIFCD subgroups, the bayesian approach suggests that higher levels of pain acceptance co‐occurred with lower levels of perceived pain. By contrast, pain catastrophizing was increased in the same manner as depression and anxiety in the dystonia wales cohort, in both the cluster analysis and using the bayesian approach, suggesting that this form of pain interpretation is more typical in those with more pronounced psychiatric symptoms. These results go some way to suggesting that the experience and interpretation of pain in those with AOIFCD may involve distinct underlying mechanistic pathways. Interestingly, previous electrophysiological studies within this clinical group have also identified changes in neuronal pathways linked with pain perception and interpretation, including abnormal inhibition (Tinazzi et al., 2019), disrupted somatosensory processing (Tinazzi et al., 2003, 2012), and alterations to the processing of painful stimuli (Lobbezoo et al., 1996). These potentially indicate that while pain itself may be secondary to the motor symptoms, some aspects of the interpretation and processing of these symptoms may be related to the underlying pathophysiological pathways involved in AOIFCD. This is also reflected in the dystonia coalition cohort where there is no significant difference between the pain scores between the two identified AOIFCD subgroups after correction for multiple comparisons. The only sub‐category of pain that tended towards significance was disability due to pain, with pain duration and severity being comparable between groups. This suggests that although the pain experienced may not differ between AOIFCD subgroups, there may be a difference between how the pain is perceived and interpreted.

One of the advantages of the bayesian mixed modelling approach is that it not only allows for missing data in the context of multiple clinical assessments, but also provides an indication of the minimum clinical dataset needed to determine phenotypic subgroups, important in the development of future multi‐national collaborations as well as more personalized medicine approaches. Similar approaches have been used in other neurological disorders, successfully combining clinical and biological data, to determine the predominant phenotypic axes linked with individual disorders (Fiford et al., 2020; Iddi et al., 2018). This study suggests that future phenotypic analyses should include assessments aimed at characterizing the more commonly assessed psychiatric symptoms (anxiety, depression, and OCD), alongside more detailed subtyping of anxiety‐related symptoms, notably social anxiety. Assessment of pain symptoms should include an indication of pain severity, the degree to which this is accepted and the more psychological responses to pain, such as, catastrophizing. Our results would indicate that inclusion of all of these components would allow for phenotypic subgrouping, potentially of importance in the targeting of treatment in future clinical trials.

Although the two AOIFCD cohorts examined demonstrate similar characteristics, there are some differences that should be noted. Higher levels of QoL impairment were observed in the dystonia wales cohort, as well as older age at examination and longer disease duration. However, disease duration did not show a strong association with any of the phenotypic axes derived, nor a strong relationship with QoL suggesting that other factors may be contributing to this lower reported level of QoL. Distinct geographical locations of recruitment, cultural differences, variation in healthcare services, and care provision, may be some of the factors accounting for these differences.

The differences in symptom type and methods (in person vs. online) of data collection formed the main limitations of this study. While this presents some challenges in allowing for direct comparison between the two groups, we have sought to use analytical methods that allow for examination of symptom groups rather than specific questionnaires. Our bayesian mixed model approach also allowed for some refinement of those areas of phenotyping that might be most relevant in future comprehensive non‐motor phenotyping studies, potentially allowing for greater alignment in investigation between future collaborative initiatives and research groups.

The low number of participants that had complete datasets, and therefore qualified for the cluster analysis is also a limitation of this study. This was due to the self‐report nature of the data collection in the dystonia wales cohort. In the dystonia coalition cohort, there were a number of participants who also did not complete all the assessments. Further, ascertainment bias may have been introduced as all participants were recruited from specialist neurology clinics, however, this increases the likelihood of accurate diagnosis. Accurate diagnosis was particularly important for this study as we were investigating variation within a specific diagnosis, therefore contamination with other diagnoses could invalidate our results. With these factors in mind, these results should be interpreted with caution, although the use of two analytical methods to determine potential non‐motor subgroups and phenotypic variation within this clinical group overcomes this to some degree.

Data on treatment, including treatment with botulinum toxin, was not available in the dystonia coalition cohort and therefore, for consistency was not included in any of these analyses. We recognize that this is likely to be an important feature and may impact on variation of non‐motor symptoms. Future work should include data on treatment to determine the impact it may have on non‐motor phenotypic subgroups within AOIFCD.

Using detailed non‐motor phenotyping, two main symptomatic subgroups have emerged within cohorts of individuals diagnosed with AOIFCD, one comprising approximately a third of individuals and exhibiting prominent psychiatric symptomatology, with increased sleep impairment and tendency towards pain catastrophizing, while the remaining two thirds of individuals show much lower levels of these symptoms. Symptoms in the former group appear to have a greater impact on reducing QoL, suggesting that recognition and management of the psychiatric symptom spectrum are key treatment components. Further work will involve replication of these findings in larger cohorts together with their analysis in the context of biological data in order to begin to explore whether these phenotypic subgroups are the consequence of distinct underlying pathophysiological mechanisms.

### PEER REVIEW

The peer review history for this article is available at https://publons.com/publon/10.1002/brb3.2292.

## CONFLICT OF INTEREST

The authors state that they have no relevant conflicts of interest to report.

## AUTHOR CONTRIBUTIONS

*Study concept and design*: Zehra Yilmaz and Kathryn J. Peall. *Data acquisition and processing of the Dystonia Wales dataset*: Megan E. Wadon, Grace A. Bailey, Zehra Yilmaz, Emily Hubbard, Meshari AlSaeed, Amy Robinson, Duncan McLauchlan, Kathryn J. Peall. *Data acquisition and processing of the Dystonia Coalition dataset*: Richard L. Barbano, Laura Marsh, Stewart A. Factor, Susan H. Fox, Charles H. Adler, Ramon L. Rodriguez, Cynthia L. Comella, Stephen G. Reich, William L. Severt, Christopher G. Goetz, Joel S. Perlmutter, Hyder A. Jinnah. *Statistical Analysis*: Megan E. Wadon, Katharine E. Harding, Cynthia Sandor, Kathryn J. Peall. *Drafting a significant portion of the manuscript and preparing the figures*: Megan E. Wadon and Kathryn J. Peall.

## Data Availability

The data that support the findings of this study are available from the corresponding author upon reasonable request.
